# Role of Female Research at the Asociacion Mexicana de Cirugia General Annual Meeting: A Retrospective Analysis From 2013 to 2019

**DOI:** 10.3389/fsurg.2022.900076

**Published:** 2022-05-13

**Authors:** Lorelí Mejía-Fernández, Fernanda Romero-Hernández, Ana López-Ruiz, Fidel Lopez-Verdugo, Jorge Sanchez-Garcia, Jose L. Martinez-Ordaz, Eduardo Moreno-Paquentin, Elena Lopez-Gavito

**Affiliations:** ^1^ Tecnológico de Monterrey, Escuela de Medicina y Ciencias de la Salud, TecSalud, Monterrey, Nuevo León, México; ^2^ Department of Surgery, University of California, San Francisco, CA, United States; ^3^ University of California, Los Angeles, David Geffen School of Medicine, United States; ^4^ Hepatobiliary Surgery and Transplant Services, Intermountain Medical Center, Salt Lake City, UT, United States; ^5^ Department of General and Gastrointestinal Surgery, UMAE Hospital de Especialidades – Centro Médico Nacional Siglo XXI (Instituto Mexicano del Seguro Social, IMSS), Mexico City, Mexico; ^6^ Department of Surgery, Centro Medico ABC, Mexico City, Mexico; ^7^ Department of Surgery, Hospital Sharp Mazatlan, Mazatlan, Mexico

**Keywords:** gender role, academic surgery, surgical training, general surgery, inclusion, surgeon, leadership, bias

## Abstract

**Background:**

Academic surgery has been a traditionally male-dominated field. Female contribution remains challenging. In Mexico, there is no published evidence regarding gender disparity in academic surgery. We aimed to analyze the female role in clinical research submitted to the Asociación Mexicana de Cirugía General (AMCG).

**Methods:**

Retrospective study evaluating abstracts submitted to AMCG annual meetings from 2013 to 2019. Categorical variables were compared using χ^2^ test. Univariate logistic regression was performed to calculate odds ratios (OR) followed by a log-binomial logistic regression model to obtain the adjusted relative risk (aRR) for acceptance as an oral presentation.

**Results:**

Overall, 7,439 abstracts were analyzed of which 24.2% were submitted by females. Female-submitted abstracts increased from 22.5% to 25.3% during 2013–2019 (*p* = 0.15). The proportion of 47 abstracts submitted by females was higher in the resident group (27.7% vs. 18.8%; *p* < 0.001). The percentage of females’ abstracts selected for oral presentation was less than the percentage of males’ 49 abstracts selected for presentation (9% vs. 11.5%; *p* = 0.002). Females’ abstracts submitted have a 50 23.5% decreased chance of being selected for oral presentation (OR = 0.765, CI 95%, 0.639–0.917, 51 *p* = 0.003). However, after adjusting for research type and trainee status, the gender of the oral 52 presenting author showed no association (aRR = 0.95, CI 95%, 0.8–1.1, *p* = 0.56).

**Conclusion:**

In Mexico, the female role in academic surgery is still limited. These results should 55 encourage professors and program directors to identify and address factors contributing to gender 56 disparities.

## Introduction

In the last half-century, female enrollment in medicine has increased significantly. In 2016, females accounted for 47% of medical school graduates worldwide (1, 2). In Mexico, 53% of medical graduates were females during the last decade (3). Despite this increase, gender disparity remains a constant issue in the areas of promotions, remunerations, evaluations, and scientific publications (4–6).

Females have gained positions in traditionally male-dominated specialties such as general surgery (7). However, there is still a wide disparity favoring males. In general surgery, females represent only 43% of residents in the United States and only 22% are active physicians (8, 9). In Mexico, only 22% of general surgery residents are females (3). Furthermore, females represent 16% of active members of the Asociación Mexicana de Cirugía General (AMCG).

The enrollment of women in academic surgery continues to be a challenge as well. According to the AAMC, women represent 38% of full-time academic faculty, 21% of full professors and 15% of department chairs. However, women represent less than 20% of full-time surgical faculty, less than 10% of full professors of surgery and only 5.7% of surgical chairs (7, 10). In the Surgery Department of the National Autonomous University of Mexico (UNAM), only 22% of the professors are women (11). The first female did not get a seat on the Executive Board in the AMCG until 1990. After 19 years, the first female President of the AMCG was elected and the first Executive President in 2017 (11). Likewise, the National Medical Academy in Mexico elected its first female President in 2019. As a result, it is not a surprise that the number of females in surgery research is considerably lower than males.

Currently, there is no published data regarding female role in surgical research in Mexico. Annually, the AMCG organizes scientific meetings and encourages research in surgery by calling for abstract submission. The information provided by submitted abstracts to this meeting can be an indirect measure of the scientific activity among surgical residency programs and academia in Mexico. The aim of this study was to evaluate the female role in surgical clinical research performed in Mexico by analyzing submitted abstracts to the AMCG.

## Materials and Methods

Submitted abstracts to AMCG annual meetings were retrospectively analyzed from 2013 to 2019. Databases were provided by the AMCG and included information regarding presenting author, acceptance status, type of presentation, design of the study, topic classification according to surgical subspecialty, and trainee status. Submitted abstracts went through a blinded peer-reviewed process. The author’s name, trainee status, and institution were not available during the acceptance/rejection decision making. For this reason, any differences observed in female representation were unlikely to have been caused by potential biases in abstracts reviewers. Submitted abstracts during the study period were screened for inclusion in the study. Multiple abstracts (*n*) submitted by the same individual were included as *n* observations. Abstracts accepted for video sessions and those submitted as “Video/surgical technique” were excluded from the analysis.

Sex of presenting author was assigned independently by four authors (FRH, ALR, LMF, and JSG) using a binary system (i.e., female or male), as previously reported (12, 13). A three-tiered approach was used: (1) determination of sex using traditional naming conventions; (2) search of presenting authors using the association’s members directory; (3) internet search of presenting author’s name and institution. Cases in which the presenting author’s sex could not be determined by the above-mentioned steps were excluded from the analysis.

Confidentiality of authors was respected according to the terms and conditions signed and consent provided at the moment of abstract submission.

### Statistical analysis

Descriptive results are reported as percentages. Categorical variables were compared using χ2 test to determine differences between groups. Univariate logistic regression was performed to calculate odds ratios (OR) and identify any associations among analyzed variables. Statistically significant variables in univariate analysis were included in a log-binomial logistic regression model to calculate adjusted relative risks (aRR). All statistical tests were two-tailed, and *p* values <0.05 were considered significant. Statistical analysis was performed using R software version 3.6.2 (R Core Team, 2019; Supplementary file).

## Results

**Figure 1:**
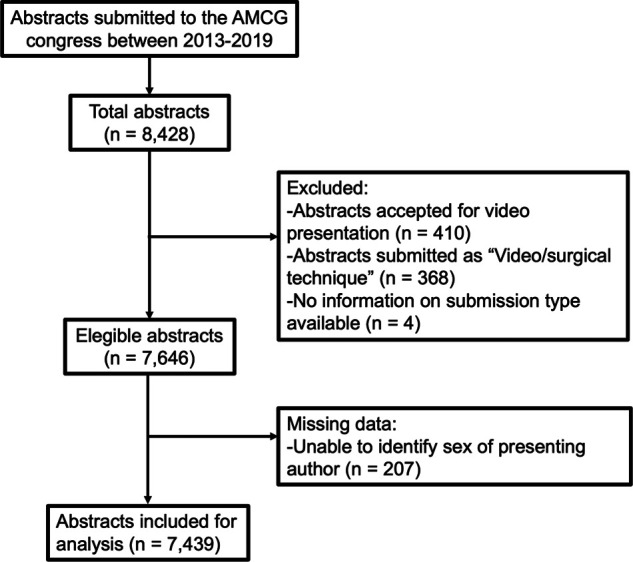
Flow diagram showing data obtained for this study.

**Figure 2:**
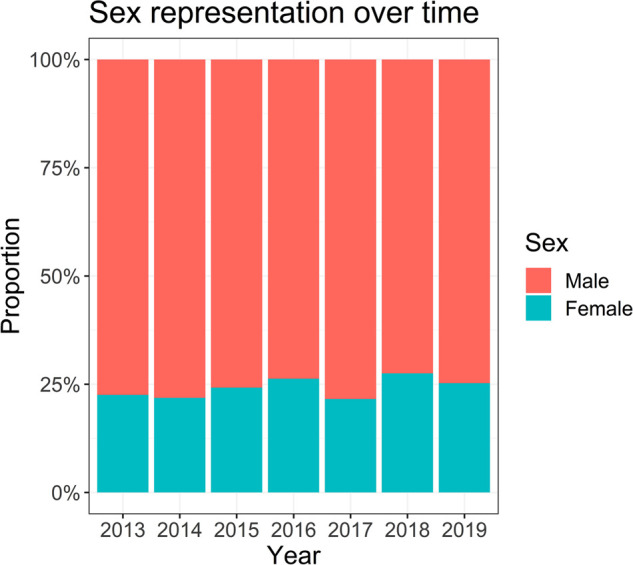
Abstracts submitted to AMCG annual meetings from 2013 to 2019 compared by sex. The year with the highest proportion of accepted abstracts from females was 2018, while 2017 showed the lowest proportion.

A total of 8,428 abstracts were submitted between 2013 and 2019, and 7,439 were ultimately included in this analysis (Figure 1). A total of 6,017 abstracts were accepted for presentation (809 for oral presentation and 5,208 for poster presentation) while 1,422 were rejected. Overall, 24.2% (*n* = 1,806) were submitted by females. Abstract submissions by gender were accepted at a similar rate (female 82% vs*.* male 80.5%; *p* = 0.174). The percentage of abstracts submitted by females increased from 22.5% in 2013 to 25.3% in 2019 (Figure 2), although this increase was not statistically significant (*p* = 0.15).

### Trainee status

When comparing males and females by trainee status, the percentage of abstracts submitted by females in the resident group was 27.7%, while the non-resident group was 18.8%. This difference between the resident and non-resident group compared by sex was statistically significant (*p *< 0.001). We did not have enough information about trainee status to trace a progression during the analyzed period.

### Topic

**Figure 3:**
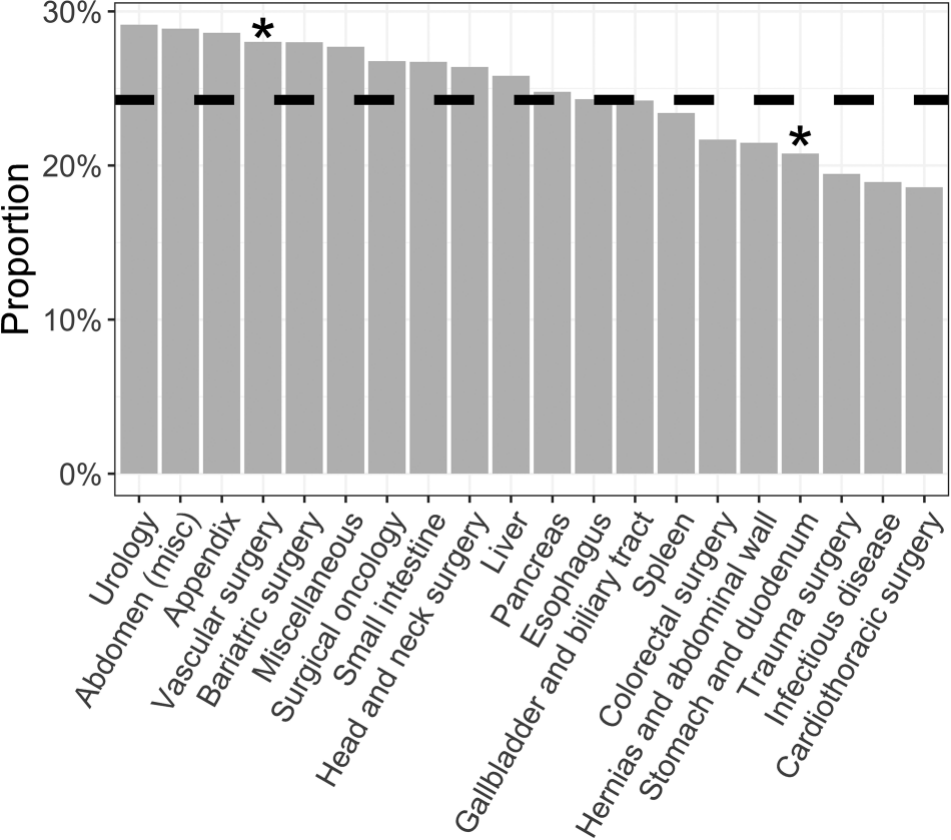
Abstracts submitted by females stratified by topic/subspecialty. The dashed line represents the mean female proportion (24.2%). * *p* < 0.05

The proportion of females varied across the different surgical topics (Figure 3). Among the top 20 most frequent abstract topics, females had the highest representation in pediatric surgery, followed by transplant surgery and urology. The lowest representations were found in cardiothoracic surgery, minimally invasive surgery, and experimental surgery/surgical research. However, the only topics in which females were significantly over and underrepresented were miscellaneous and infectious diseases, respectively.

### Analysis per Study Design, Trainee Status, Author’s Sex and Status of Acceptance

**Figure 4:**
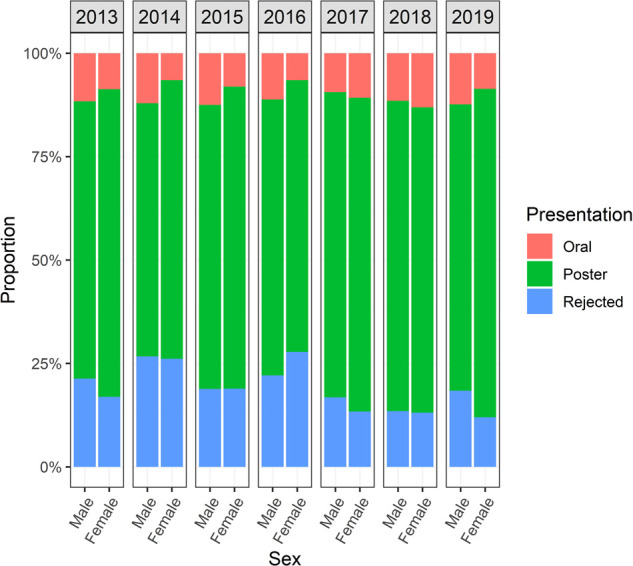
Comparison of abstracts submitted to AMCG annual meeting from 2013 to 2019 by sex and status of acceptance (oral, poster, or rejection).

**Figure 5:**
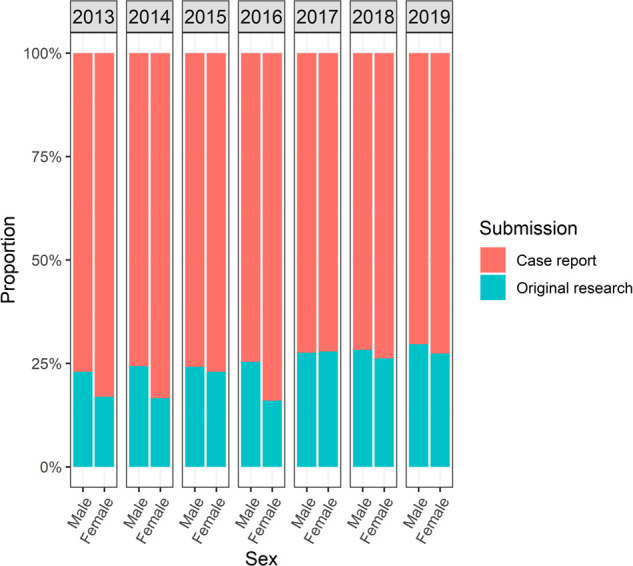
Comparison of abstracts accepted to AMCG annual meeting from 2013 to 2019 by sex and study design (case report, original research).

Throughout the study period, the percentage of original research abstracts submitted by females increased from 16.9% in 2013 to 27.3% in 2019 (*p* < 0.01). A smaller percentage of abstracts submitted by females was selected for oral presentation compared to those by males (9% vs*.* 11.5%; *p* = 0.002, Figure 4). Overall, abstracts submitted by females that were classified as original research represented 22.4%, compared to 26.3% of those by males (*p* = 0.001, Figure 5).

In a subgroup analysis, females in the resident group had fewer original abstracts (23.1% vs. 34.9%, *p *= 0.0001), fewer abstracts selected for oral presentations (8.6% vs. 13.7%, *p *= 0.02) but similar abstracts rejected (14.5% vs. 12.9%, *p *= 0.6) when compared to females in the non-resident group. Similarly, when comparing males in the resident group *versus* the non-resident group, the former had fewer original abstracts (22.9% vs. 35.4%, *p *< 0.0001), fewer abstracts selected for oral presentations (8.6% vs. 15.4%, *p *< 0.0001) and fewer abstracts rejected (15.8% vs. 19.7%, *p *= 0.004)

Compared to males, unadjusted logistic regression showed that abstracts submitted by females had a 23.5% decreased chance of being selected for oral presentation (OR = 0.765, CI 95%, 0.639–0.917, *p* = 0.003). However, this association was no longer statistically significant when adjusted for the type of research and trainee status (aRR = 0.95, CI 95%, 0.8–1.1, *p *= 0.056, Supplementary Table S1).

Original research studies were more likely to be accepted for oral presentations (aRR = 136.7, CI 95%: 70.9–263.5, *p *< 0.001), while abstracts submitted by surgery residents had a decreased tendency of being accepted as oral presentations (aRR = 0.87, CI 95%: 0.75–1, *p *= 0.058). In the adjusted logistic regression, oral presentations and resident status remained statistically significant after excluding the rejected abstracts (Supplementary Table S2).

## Discussion

Gender disparity among surgical specialties is a current concern that negatively impacts the professional development of females. Overall, submitted abstracts that had a female as a first-author accounted for 24.2%. Females’ participation in surgical clinical research showed a 2.8% increase in seven years but did not reach statistical significance. Jagsi *et al.* reported a 5-fold increase of females participating as first or senior authors among original high-quality medical research publications in a 35-year period (14). It is important to notice that surgical journals had the lowest increase in female participation compared to journals related to other medical disciplines such as obstetrics & gynecology and pediatrics. More recently, Mueller *et al.* reported that females in academic surgery had significantly lower H-index and publications compared to males (15). Our data indirectly indicates gender disparity in academic surgery research in Mexico similar to these reports.

Several factors may be contributing to the differences shown in this study regarding abstract submission rates by females enrolled in a surgical residency program. Surgery residency admission in Mexico is complex, demanding, and limited with an acceptance rate of 20% per year (16, 17). In the last 7-years, from all applicants accepted for surgery residency, the proportion of females has been 22 ± 1% (3). (Supplementary Figure S1). In the United States, the female percentage in surgical training programs was nearly 40% in 2013 (18). Despite the fact that about half of Mexican medical school graduates are females, there has not been an equitable increase in the proportion of females enrolling in surgery specialty programs in Mexico (16, 19). This limited enrollment directly impacts the proportion of females actively participating in surgical research. Therefore, there is a need to address the factors leading to the low enrollment of females in surgical residency programs after graduation from medical school. In Mexico, medical students have reported that their specialty choice is highly dependent on their experience during medical school, and 24% apply to general surgery programs (17). In Latin America, only 8.6% of females intend to pursue general surgery training and are 22% less likely to choose this field compared to males (19). Globally, male medical students are more likely to choose surgery or orthopedics residencies compared to females (4). This decision is mainly driven by the potential for work-life balance and, particularly in females, by the presence of hostility and sexism within the residency environment (20). It has been reported that medical students perceive surgical residency programs to be discriminatory and prone to abuse and burnout (21–23). Mistreatment perceptions have also been reported by general surgery residents (24).

While sparse, our results are similar to previous studies in other countries of Latin America. In a recent study, Bueno Motter et al. investigated women’s representativeness across surgical departments of Brazilian universities, Brazilian surgical societies, and speakers in surgical events (25). They found that of university departments, only 11.2%were women and only three universities had women as department chairs. Also, in surgical societies, only 8.6% of positions were held by women. When analyzing speakers’ participation in surgical events, only 13.3% of 6686 speakers were women. Similarly, Sarmiento Altamirano et al. surveyed a total of 105 women surgeons to evaluate the current representation of women surgeons in Ecuador (26). Of the female surgeons surveyed, 67% reported that leadership in their workplace, both departmental and hospital levels tended to be led by males, and only 6.7% were occupied by females. These results should serve as an overall status of gender disparities in Latin America to promote changes towards a more equal representation.

### Trainee Status

Abelson *et al.* reported that female participation in General Surgery has increased from 20% to 40% during the last 20 years in the United States (27). Despite the fact that only 22% of Mexican surgery residents are females, our study showed an overrepresentation of female residents submitting abstracts (27%) (28). Although the factors contributing to greater participation of female residents remain to be elucidated, this might suggest that, once, outside the residency program, women encounter more obstacles that hinder their continued participation in research. Alternatively, this might suggest a higher interest in female residents to participate in surgical research. It’s important to consider that women face additional challenges during residency, such as pregnancy and motherhood, exacerbating the research gap. While no research on the number of women who get pregnant during general surgery residency is available, from the author’s experience we can say that pregnancy is not common during surgery residency in Mexico. This could be related to several factors including limited monetary compensation to raise a family, fear of losing their residency status, and lack of appropriate and supporting maternity leave policies. Definitely, this should be a significant area of opportunity for future research to approach gender disparities, modify current policies and improve current residency programs.

The non-resident group may involve board-eligible female surgeons, medical students, and/or other healthcare professionals. From the authors’ personal experiences, the number of medical students participating as presenting authors in the AMCG meeting is very low due to the lack of research curriculum, tutoring and funding in the majority of medical schools. Thus, we think that this group is mostly represented by senior academic surgeons, which may be supported by the fact that the non-resident group had more original abstracts and a greater number of their abstracts selected for oral presentation compared to the resident group. Even though it is difficult to analyze this group, we can hypothesize that multiple factors influence the decreased research participation by non-residents, including the low representation of female surgeons, family and personal commitments, or academic requirements (8). Further research is imperative to determine the cause for lower abstract submission by female non-residents.

### Topic and Specialties

Females had higher participation in urology, followed by abdominal, appendix, and bariatric surgery (Figure 3). Contrary to what previous studies have shown, our data display more female participation in traditionally male-dominated surgical subspecialties (29, 30). Furthermore, our study showed that the lowest female representation accounted for cardiothoracic surgery and infectious diseases related abstracts, supporting previously published data (27, 31). Valsangkar et al. showed the highest gender disparity in publications related to surgical subspecialties, such as in acute care surgery, surgical oncology, vascular surgery, plastic surgery, and cardiac surgery (32). Differences observed within different topics should be interpreted cautiously, as the AMCG is a general surgery meeting. It is well known that gender impacts the choice of subspecialty, in that males are more likely to enter a fellowship (70% vs. 43%), and females tend to select fellowships that are less time-demanding and provide more lifestyle flexibility (29). Overall, there is a tendency for females to choose specialties that favor an optimal work-life balance. For instance, an increased female enrollment has been reported in subspecialties, such as critical care surgery and colorectal surgery (27).

### Study Design and Acceptance Status

Even though the rate of abstract acceptance between females and males is similar, we describe a significantly smaller proportion of female abstracts being selected for oral presentation. Adjusted multivariable analysis revealed that the design of study and trainee status, rather than gender, are the most important factors for an abstract to be accepted for oral presentation.

Similarly, the 2018 Annual Meeting of the Society of Thoracic Surgeons in the United States reported that only 12.9% of oral abstracts were presented by females (31). In addition, females represented 19.4% of plenary speakers, 29% of plenary and keynote speakers, and 28.5% of speakers in American Surgical Conferences, United States Medical Education Conferences and Canadian Anesthesiologists’ annual meetings, respectively (12, 13, 33).

Research quality is strongly related to research funding. In the United States, male faculty receive greater proportions of larger NIH grants (32). In Mexico, females comprise only 15% of surgery scientists registered at the National System of Researchers (SNI). SNI is commonly considered the cornerstone of scientific promotion and funding support in the country (34). This could be one of the factors contributing to the low number of original research abstracts submitted by females. However, there is limited data on research funding in Mexico.

Factors contributing to the professional gender gap have been described elsewhere (4, 10). Academic factors include early exposure to positive role models, effective mentorship, rough training environments, harassment, remuneration gap, and inclusion in high-quality research studies. Female surgery residents tend to receive less mentorship compared to males (35). Heath *et al.* reported that female trainees’ evaluations are more likely to include emotive terms (e.g., empathetic, delight, warm), as opposed to their male counterparts who are often described with ability (e.g., master, complexity) and research (e.g., trials, studies, data) terms in their evaluations (36).

Personal and social factors such as professional satisfaction, time commitment, lifestyle, and family planning can influence the development of a female resident or medical student (2, 4, 10, 21–23). For instance, Seemann *et al.* found up to 56% gender discrimination rates in female surgeons; however, the mean score of career satisfaction in these women was 8.6 (scale 1–10) (2). Schwarz et al. reported similar mean work satisfaction scores between female (69.5%) and male (75.7%) surgeons (37). Despite the fact that females have more opportunities nowadays than in the past, much remains to be done. The so-called “leaky pipeline” phenomenon demonstrates that females are less likely to have a full-professor status, even after accounting for scientific productivity (38–40). If this trend is allowed to continue its course, gender parity in academic ranks would not be achieved until 2136 (27).

It seems that social role expectations keep playing a role in achieving balance between professional and personal life. Implicit and explicit gender biases exist in healthcare professionals, who often associate males with professional development, whereas females are more likely to be associated with family and family medicine (41). Gender bias and stereotypes affect career engagement and technical performance among those pursuing a career in academic surgery (42). Indeed, academic and social factors impact academic surgery in Mexico. However, there is not enough data from Mexico describing the different factors that affect or influence a female’s decision to engage in research projects during or after residency. This could be an area of opportunity for future research.

Several associations have already developed tools and resources to identify detrimental factors, such as sexual discrimination. For instance, The National Academies of Sciences, Engineering, and Medicine developed a consensus to evaluate sexual harassment in females (43). Furthermore, the University of Louisville has also implemented changes to ensure teamwork and non-discriminatory environments (29). These tools, among others, could help point out specific factors that can be acted upon to enhance the scientific development of females.

### Limitations

This study has some limitations, aside from those inherent in its retrospective nature. First, overall female participation could not be assessed as we analyzed only the sex of the presenting authors, and the role of co-authors could disclose additional findings, especially when looking at senior authors. We decided to focus on presenting authors because we felt it to be a reliable marker of research participation, as presenting authorship is usually granted to the greatest contributor. In Mexico, it is not a universal practice to place the senior author as the last co-author; this prevented us from assessing gender disparities in this group. It should be noted that only abstracts submitted to the AMCG meetings were analyzed, which limits the generalizability of our results. However, as the largest academic surgery platform in Mexico, AMCG meetings represent an overall picture of the status of academic surgery in our country.

As a social phenomenon, analysis of longer periods of time may be needed to better identify changes in gender discrimination. However, due to data availability, this study only included the last seven years. Although sex and gender are used interchangeably, these represent different dimensions. Due to the retrospective nature of the study, the gender identity of presenting authors could not be collected. As gender is a social construct based on expected roles and behaviors in society, differences in gender identity in academic surgery could uncover results that may have been overlooked by our sex-based analysis.

## Conclusion

This study showed that in Mexico, the female role in academic surgery is still limited, with only a quarter of submitted abstracts to the last AMCG meetings having a female as the first author. This might be related to the lower number of females in surgery, but further research is needed. Increasing female participation in original and high-quality surgical research is crucial to start changing the *status quo* and reducing the gender gap. The low increase in females’ abstract submissions during the study period should encourage surgical educators and general surgery leadership to identify and address factors contributing to gender disparities, beginning in the early stages of medical school and continuing throughout the entire professional careers.

## Data Availability

The original contributions presented in the study are included in the article/Supplementary Material, further inquiries can be directed to the corresponding author/s.
