# Supplementary material for: Role of Female Research at the Asociacion Mexicana de Cirugia General Annual Meeting: A Retrospective Analysis From 2013 to 2019
Source: Front Surg. 2022 May 13;9:900076. doi: 10.3389/fsurg.2022.900076 (PMC9406406; doi:10.3389/fsurg.2022.900076)
Supplement: Supplementary file 1 [file Data_Sheet_1_v1.pdf]

amcg_r markdown

Descriptive analysis

Read amcg database and store it in amcg object. Identify structure of dataframe

```
setwd("~/Documents/Otros/AMCG/Manuscript")
amcg <- read_csv("amcg.csv")
```

```
## Parsed with column specification:
## cols(
##   year = col_double(),
##   presentation = col_character(),
##   sex = col_character(),
##   resident = col_logical(),
##   type = col_character(),
##   sector = col_character(),
##   topic = col_character(),
##   type_2 = col_character()
## )
```

```
glimpse(amcg)
```

```
## Rows: 8,428
## Columns: 8
## $ year         <dbl> 2013, 2013, 2013, 2013, 2013, 2013, 2013, 2013, 2013, ...
## $ presentation <chr> "Poster", "Poster", "Rejected", "Rejected", "Poster", ...
## $ sex          <chr> "Male", "Male", "Male", "Male", "Male", "Male", "Male"...
## $ resident     <lgl> NA, NA, NA, NA, NA, NA, NA, NA, NA, NA, NA, NA, NA, NA...
## $ type         <chr> "Case report", "Case report", "Case report", "Case rep...
## $ sector       <chr> "SSA", "SSA", "SSA", "SSA", "SSA", "SSA", "SSA", "SSA"...
## $ topic        <chr> "Colorectal surgery", "Colorectal surgery", "Colorecta...
## $ type_2       <chr> "Case report", "Case report", "Case report", "Case rep...
```

This dataframe includes all submitted abstracts betwee 2013-2019. Abstracts accepted for a video presentation or submitted as video/surgical technique will be excluded from the analysis. Cases where sex of presenter could not be determined are excluded from the analysis.

```
sum(amcg$presentation == "Video")
```

```
## [1] 410
```

```
amcg <- amcg %>%
        filter(presentation != "Video") %>%
        droplevels()

sum(is.na(amcg$type_2))
```

```
## [1] 4
```

```r
amcg <- amcg %>%
        filter(type_2 != is.na(type_2)) %>%
        droplevels()

sum(amcg$type_2 == "Video/surgical technique")
```

```
## [1] 368
```

```r
amcg <- amcg %>%
        filter(type_2 != "Video/surgical technique") %>%
        droplevels()

nrow(amcg)
```

```
## [1] 7646
```

```r
sum(is.na(amcg$sex))
```

```
## [1] 207
```

```r
amcg <- amcg %>%
        drop_na(sex)
```

Convert 'sex' variable to factor

New columns are created with 1)rejection status and 2) the proportion of abstracts that were accepted for oral presentations.

```r
amcg <- amcg %>%
        mutate(rejected = presentation == "Rejected")

amcg <- amcg %>%
        mutate(oral = presentation == "Oral")

##Summary of final data
summary(amcg)
```

```
##       year        presentation      sex         resident
##  Min.   :2013   Oral     : 809   Male  :5633   Mode :logical
##  1st Qu.:2015   Poster   :5208   Female:1806   FALSE:1476
##  Median :2016   Rejected:1422                  TRUE :2927
##  Mean   :2016                                  NA's :3036
##  3rd Qu.:2018
##  Max.   :2019
##
##                        type          sector
##  Case report            :5551   SSA    :3137
##  Original research      : 766   IMSS   :1544
##  Review                 : 336   Private: 962
```

```
##  Case series          : 311   ISSSTE : 491
##  Cohort study         : 176   State  : 422
##  Cross-sectional study: 102   (Other): 861
##  (Other)              : 197   NA's   :  22
##                               topic                       type_2      rejected
##  Gallbladder and biliary tract: 795   Case report      :5551   Mode :logical
##  Small intestine              : 782   Original research:1888   FALSE:6017
##  Colorectal surgery           : 677                            TRUE :1422
##  Hernias and abdominal wall   : 502
##  Infectious disease           : 391
##  Abdomen (misc)               : 381
##  (Other)                      :3911
##     oral
##  Mode :logical
##  FALSE:6630
##  TRUE :809
##
##
##
##
```

Contingency table including sex of presenter over time:

```
addmargins(table(amcg$sex, amcg$year), 1)
```

```
##
##          2013 2014 2015 2016 2017 2018 2019
##   Male    670  711  695  859  976  764  958
##   Female  195  199  222  306  269  290  325
##   Sum     865  910  917 1165 1245 1054 1283
```

```
prop.table(table(amcg$sex, amcg$year), 2)*100
```

```
##
##              2013     2014     2015     2016     2017     2018     2019
##   Male   77.45665 78.13187 75.79062 73.73391 78.39357 72.48577 74.66875
##   Female 22.54335 21.86813 24.20938 26.26609 21.60643 27.51423 25.33125
```

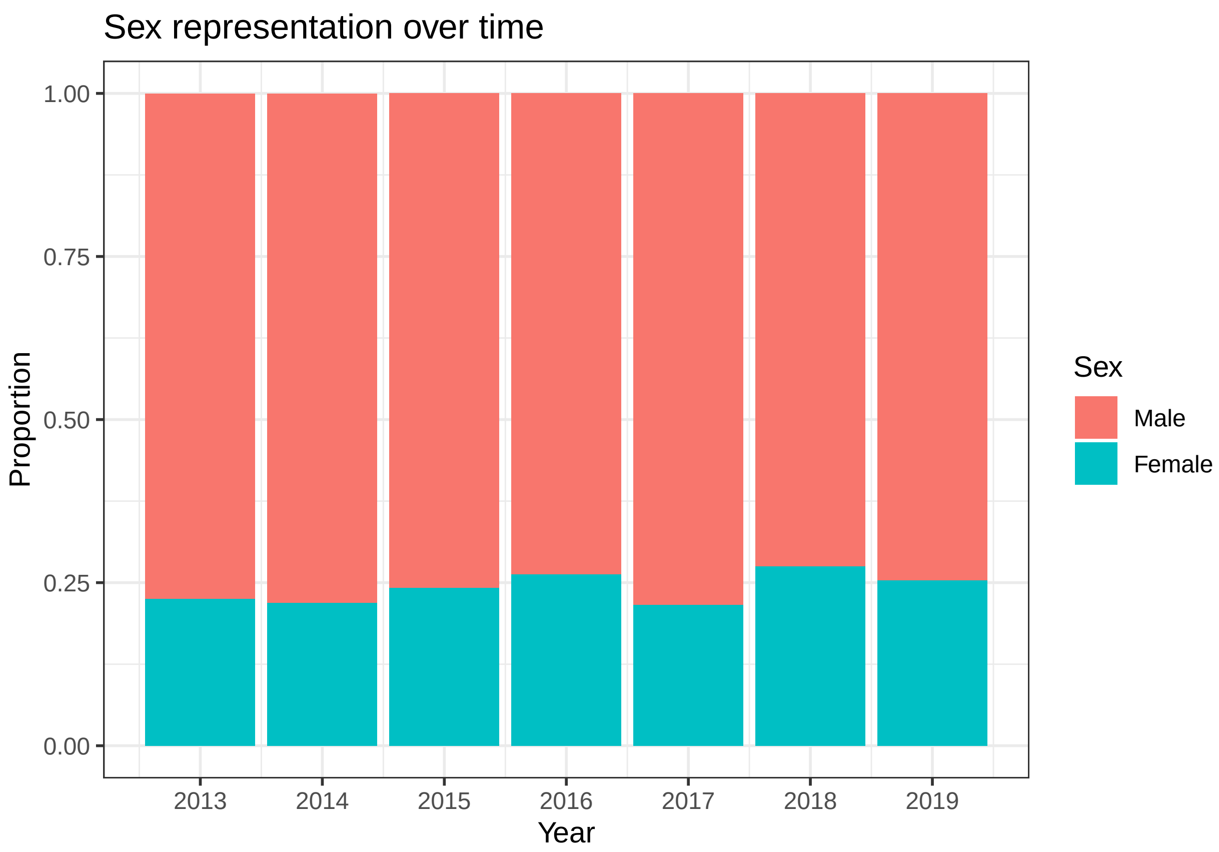

Presentation type over time by sex group

```
## `summarise()` regrouping output by 'year', 'sex' (override with `.groups` argument)

## # A tibble: 42 x 5
##     year sex    presentation total  prop
##    <dbl> <fct>  <fct>        <int> <dbl>
##  1  2013 Male   Oral            78 11.6
##  2  2013 Male   Poster         449 67.0
##  3  2013 Male   Rejected       143 21.3
##  4  2013 Female Oral            17  8.72
##  5  2013 Female Poster         145 74.4
##  6  2013 Female Rejected        33 16.9
##  7  2014 Male   Oral            86 12.1
##  8  2014 Male   Poster         435 61.2
##  9  2014 Male   Rejected       190 26.7
## 10  2014 Female Oral            13  6.53
## # ... with 32 more rows
```

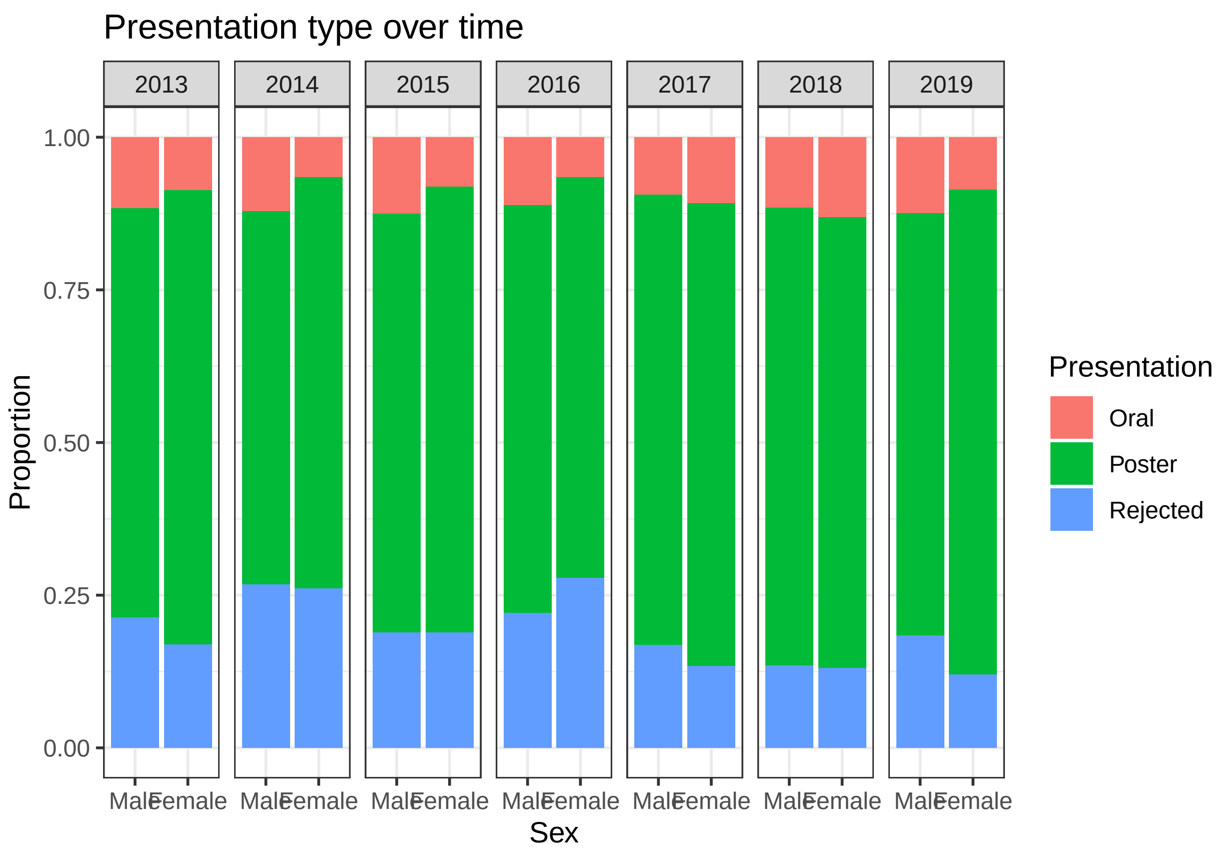

Submission type over time

```
ggplot(data = amcg, aes(x = sex, fill = type_2)) +
        geom_bar(position = "fill") +
        facet_grid(.~ year) +
        labs(title = "Submission type over time",
             x = "Sex",
             y = "Proportion",
             fill = "Submission") +
        theme_bw()
```

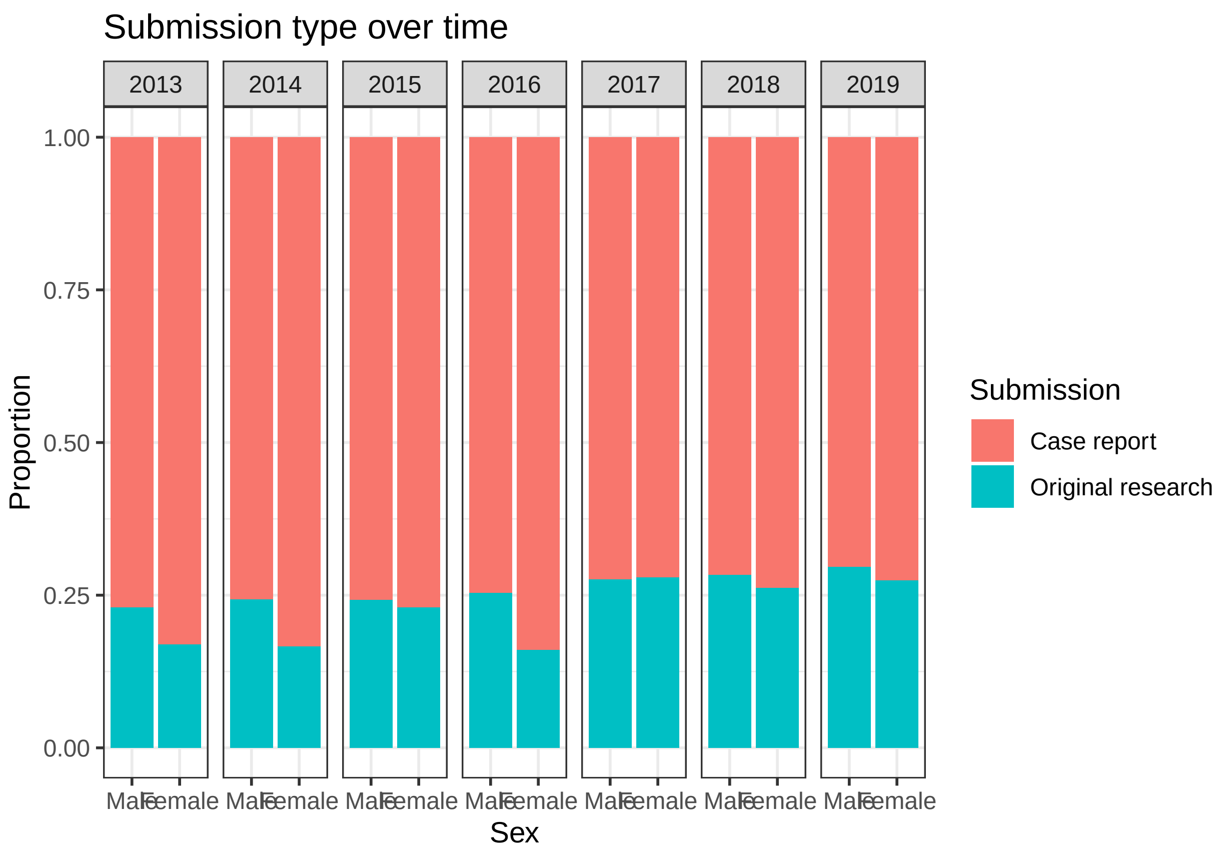

Female proportion in 20 topics with greatest number of submitted abstracts

```
top_topics <- amcg %>%
        group_by(topic) %>%
        summarize(total = n()) %>%
        arrange(desc(total)) %>%
        ungroup()
```

```
## `summarise()` ungrouping output (override with `.groups` argument)
```

```
t_20 <- top_topics$topic[1:20]

amcg_topic <- amcg %>%
        group_by(topic, sex) %>%
        summarize(total = n()) %>%
        mutate(prop = total/sum(total)) %>%
        filter(sex == "Female" & topic %in% t_20) %>%
        arrange(desc(prop)) %>%
        ungroup()
```

```
## `summarise()` regrouping output by 'topic' (override with `.groups` argument)
```

```
fem_prop <- amcg %>%
        group_by(sex) %>%
        summarize(total = n()) %>%
```

```
        mutate(prop = total/sum(total)) %>%
        filter(sex == "Female") %>%
        ungroup()
```

```
## `summarise()` ungrouping output (override with `.groups` argument)
```

```
ggplot(amcg_topic, aes(x = reorder(topic, -as.numeric(prop)), y = prop)) +
        geom_col(fill = "#B7B7B5") +
        scale_y_continuous(limits = c(0, 0.5)) +
        geom_hline(yintercept = fem_prop$prop[1], linetype = "dashed") +
        theme_bw() +
        labs(title = NULL,
             x = NULL,
             y = "Proportion") +
        theme(axis.text.x=element_text(angle=60,hjust=1))
```

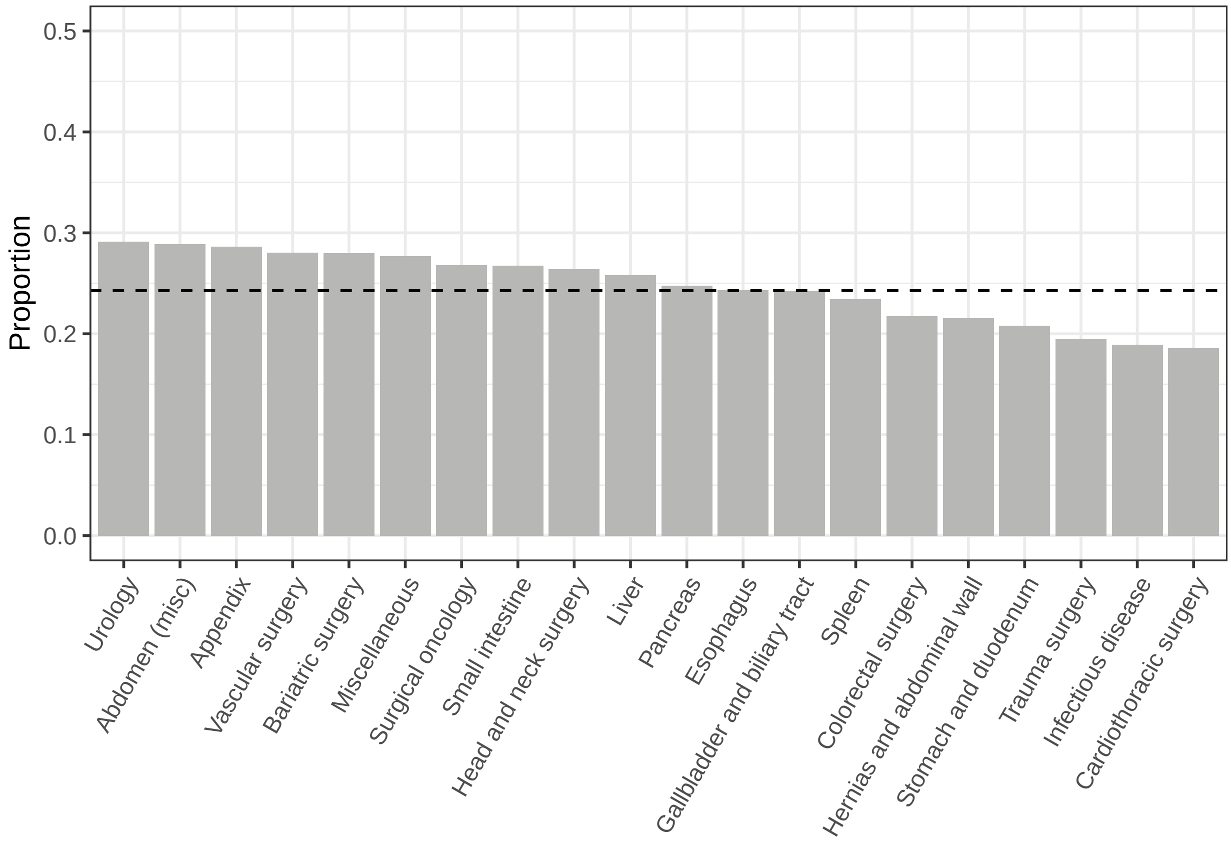

##Comparative analysis Comparison of female proportion between 2013 and 2019

```
amcg_years <- amcg %>%
        filter(year == 2013 | year == 2019)
table(amcg_years$sex, amcg_years$year)
```

```
##
##           2013 2019
##   Male     670  958
##   Female   195  325
```

```r
prop.table(table(amcg_years$sex, amcg_years$year), 2)*100
```

```
##
##              2013     2019
##   Male   77.45665 74.66875
##   Female 22.54335 25.33125
```

```r
chisq.test(amcg_years$sex, amcg_years$year)
```

```
##
##  Pearson's Chi-squared test with Yates' continuity correction
##
## data:  amcg_years$sex and amcg_years$year
## X-squared = 2.0393, df = 1, p-value = 0.1533
```

Female proportion by high and low topics

```r
chisq.test(amcg$sex, amcg$topic == "Urology")
```

```
##
##  Pearson's Chi-squared test with Yates' continuity correction
##
## data:  amcg$sex and amcg$topic == "Urology"
## X-squared = 1.3997, df = 1, p-value = 0.2368
```

```r
chisq.test(amcg$sex, amcg$topic == "Abdomen (misc)")
```

```
##
##  Pearson's Chi-squared test with Yates' continuity correction
##
## data:  amcg$sex and amcg$topic == "Abdomen (misc)"
## X-squared = 4.3504, df = 1, p-value = 0.037
```

```r
chisq.test(amcg$sex, amcg$topic == "Appendix")
```

```
##
##  Pearson's Chi-squared test with Yates' continuity correction
##
## data:  amcg$sex and amcg$topic == "Appendix"
## X-squared = 3.5438, df = 1, p-value = 0.05977
```

```r
chisq.test(amcg$sex, amcg$topic == "Vascular surgery")
```

```
##
##  Pearson's Chi-squared test with Yates' continuity correction
##
## data:  amcg$sex and amcg$topic == "Vascular surgery"
## X-squared = 1.6179, df = 1, p-value = 0.2034
```

```r
chisq.test(amcg$sex, amcg$topic == "Small intestine")
```

```
##
##  Pearson's Chi-squared test with Yates' continuity correction
##
## data:  amcg$sex and amcg$topic == "Small intestine"
## X-squared = 2.7038, df = 1, p-value = 0.1001
```

```r
chisq.test(amcg$sex, amcg$topic == "Colorectal surgery")
```

```
##
##  Pearson's Chi-squared test with Yates' continuity correction
##
## data:  amcg$sex and amcg$topic == "Colorectal surgery"
## X-squared = 2.5122, df = 1, p-value = 0.113
```

```r
chisq.test(amcg$sex, amcg$topic == "Hernias and abdominal wall")
```

```
##
##  Pearson's Chi-squared test with Yates' continuity correction
##
## data:  amcg$sex and amcg$topic == "Hernias and abdominal wall"
## X-squared = 2.0781, df = 1, p-value = 0.1494
```

```r
chisq.test(amcg$sex, amcg$topic == "Stomach and duodenum")
```

```
##
##  Pearson's Chi-squared test with Yates' continuity correction
##
## data:  amcg$sex and amcg$topic == "Stomach and duodenum"
## X-squared = 1.945, df = 1, p-value = 0.1631
```

```r
chisq.test(amcg$sex, amcg$topic == "Trauma surgery")
```

```
##
##  Pearson's Chi-squared test with Yates' continuity correction
##
## data:  amcg$sex and amcg$topic == "Trauma surgery"
## X-squared = 3.101, df = 1, p-value = 0.07825
```

```r
chisq.test(amcg$sex, amcg$topic == "Infectious disease")
```

```
##
##  Pearson's Chi-squared test with Yates' continuity correction
##
## data:  amcg$sex and amcg$topic == "Infectious disease"
## X-squared = 6.1258, df = 1, p-value = 0.01332
```

```r
chisq.test(amcg$sex, amcg$topic == "Cardiothoracic surgery")
```

```
##
##  Pearson's Chi-squared test with Yates' continuity correction
##
## data:  amcg$sex and amcg$topic == "Cardiothoracic surgery"
## X-squared = 3.0036, df = 1, p-value = 0.08308
```

Rejection rates by sex

```r
table(amcg$rejected, amcg$sex)
```

```
##
##         Male Female
##   FALSE 4536   1481
##   TRUE  1097    325
```

```r
prop.table(table(amcg$rejected, amcg$sex), 2)
```

```
##
##              Male    Female
##   FALSE 0.8052547 0.8200443
##   TRUE  0.1947453 0.1799557
```

```r
chisq.test(amcg$rejected, amcg$sex)
```

```
##
##  Pearson's Chi-squared test with Yates' continuity correction
##
## data:  amcg$rejected and amcg$sex
## X-squared = 1.8402, df = 1, p-value = 0.1749
```

Comparison of presentation type by sex

```r
cont_sex_pres <- table(amcg$sex, amcg$presentation,
                       dnn = c("Sex", "Presentation"))
cont_sex_pres
```

```
##         Presentation
## Sex       Oral Poster Rejected
##   Male     646   3890     1097
##   Female   163   1318      325
```

```r
prop.table(cont_sex_pres, 1)*100
```

```
##         Presentation
## Sex           Oral    Poster  Rejected
##   Male   11.468134 69.057341 19.474525
##   Female  9.025471 72.978959 17.995570
```

```r
chisq.test(cont_sex_pres)
```

```
##
##  Pearson's Chi-squared test
##
## data:  cont_sex_pres
## X-squared = 12.072, df = 2, p-value = 0.002391
```

```r
chisq.test(cont_sex_pres)$residuals
```

```
##         Presentation
## Sex            Oral     Poster   Rejected
##   Male    1.3496456 -0.8540045  0.6163614
##   Female -2.3835855  1.5082425 -1.0885452
```

```r
mosaic(cont_sex_pres, shade = TRUE)
```

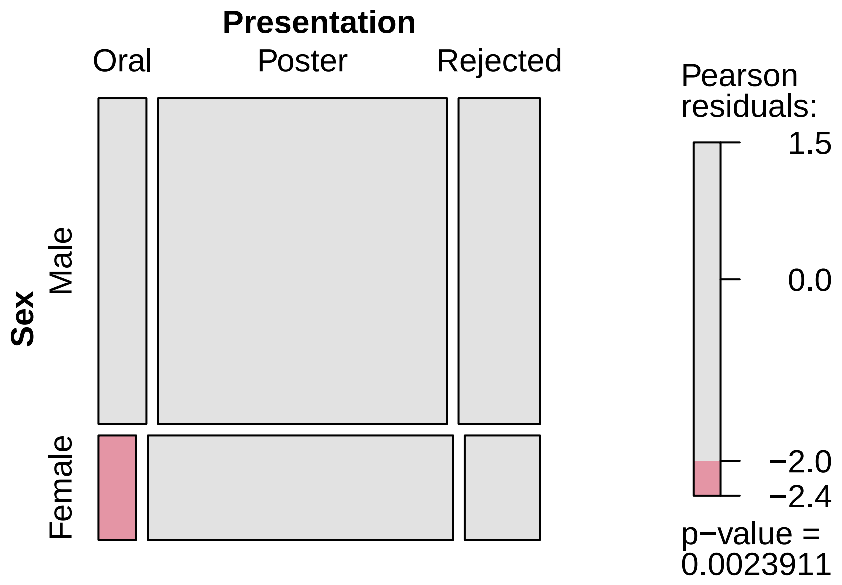

Sex representation by trainee status

```r
sex_res <- table(amcg$sex, amcg$resident,
                 dnn = c("Sex", "Resident"))

sex_res
```

```
##         Resident
## Sex      FALSE TRUE
##   Male    1198 2116
##   Female   278  811
```

```r
prop.table(sex_res, 2)
```

```
##         Resident
## Sex          FALSE      TRUE
##   Male   0.8116531 0.7229245
##   Female 0.1883469 0.2770755
```

```r
chisq.test(sex_res)$residuals
```

```
##         Resident
## Sex          FALSE      TRUE
##   Male    2.612035 -1.854859
##   Female -4.556605  3.235737
```

```r
chisq.test(sex_res)
```

```
##
##  Pearson's Chi-squared test with Yates' continuity correction
##
## data:  sex_res
## X-squared = 41.021, df = 1, p-value = 1.506e-10
```

```r
mosaic(sex_res, shade = TRUE)
```

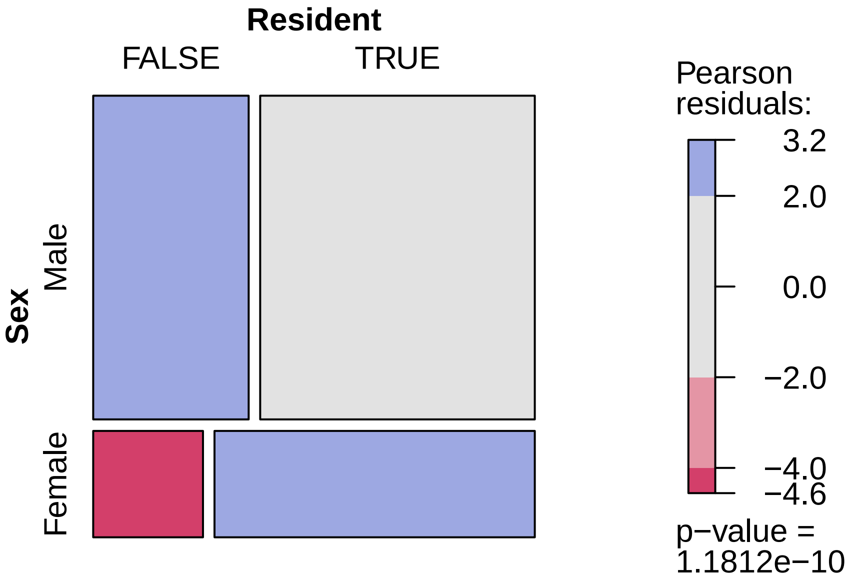

Type of abstracts submitted by sex

```
table(amcg$type_2, amcg$sex)
```

```
##
##                      Male Female
##   Case report        4151   1400
##   Original research  1482    406
```

```
prop.table(table(amcg$type_2, amcg$sex), 2)
```

```
##
##                           Male    Female
##   Case report        0.7369075 0.7751938
##   Original research  0.2630925 0.2248062
```

```
chisq.test(amcg$type_2, amcg$sex)$residuals
```

```
##                     amcg$sex
## amcg$type_2                Male     Female
##   Case report        -0.8075846  1.4262610
##   Original research   1.3847540 -2.4455899
```

```r
chisq.test(amcg$type_2, amcg$sex)
```

```
##
##  Pearson's Chi-squared test with Yates' continuity correction
##
## data:  amcg$type_2 and amcg$sex
## X-squared = 10.384, df = 1, p-value = 0.001271
```

Univariate logistic regression: oral presentation

```r
mod_oral_type <- glm(oral ~ type_2, data = amcg,
                     family = binomial)

summary(mod_oral_type)
```

```
##
## Call:
## glm(formula = oral ~ type_2, family = binomial, data = amcg)
##
## Deviance Residuals:
##    Min      1Q  Median      3Q     Max
## -1.048  -0.063  -0.063  -0.063   3.528
##
## Coefficients:
##                           Estimate Std. Error z value Pr(>|z|)
## (Intercept)                -6.2219     0.3016  -20.63   <2e-16 ***
## type_2Original research     5.9100     0.3052   19.36   <2e-16 ***
## ---
## Signif. codes:  0 '***' 0.001 '**' 0.01 '*' 0.05 '.' 0.1 ' ' 1
##
## (Dispersion parameter for binomial family taken to be 1)
##
##     Null deviance: 5116.5  on 7438  degrees of freedom
## Residual deviance: 2730.9  on 7437  degrees of freedom
## AIC: 2734.9
##
## Number of Fisher Scoring iterations: 8
```

```r
exp(summary(mod_oral_type)$coefficients[2,1] +
            qnorm(c(0.025,0.5,0.975)) * summary(mod_oral_type)$coefficients[2,2])
```

```
## [1] 202.7171 368.7170 670.6503
```

Unadjusted logistic regression: oral presentation by sex

```r
mod_oral_sex <- glm(oral ~ sex, data = amcg,
                    family = binomial)

summary(mod_oral_sex)
```

```
##
## Call:
## glm(formula = oral ~ sex, family = binomial, data = amcg)
##
## Deviance Residuals:
##     Min       1Q   Median       3Q      Max
## -0.4936  -0.4936  -0.4936  -0.4350   2.1932
##
## Coefficients:
##             Estimate Std. Error z value Pr(>|z|)
## (Intercept) -2.04379    0.04181 -48.878  < 2e-16 ***
## sexFemale   -0.26674    0.09214  -2.895  0.00379 **
## ---
## Signif. codes:  0 '***' 0.001 '**' 0.01 '*' 0.05 '.' 0.1 ' ' 1
##
## (Dispersion parameter for binomial family taken to be 1)
##
##     Null deviance: 5116.5  on 7438  degrees of freedom
## Residual deviance: 5107.8  on 7437  degrees of freedom
## AIC: 5111.8
##
## Number of Fisher Scoring iterations: 4
```

```
exp(summary(mod_oral_sex)$coefficients[2,1] +
            qnorm(c(0.025,0.5,0.975)) * summary(mod_oral_sex)$coefficients[2,2])
```

```
## [1] 0.6393376 0.7658733 0.9174526
```

Univariate log regression for trainee status

```
mod_oral_trainee <- glm(oral ~ resident, data = amcg,
                    family = binomial)

summary(mod_oral_trainee)
```

```
##
## Call:
## glm(formula = oral ~ resident, family = binomial, data = amcg)
##
## Deviance Residuals:
##     Min       1Q   Median       3Q      Max
## -0.5724  -0.5724  -0.4243  -0.4243   2.2146
##
## Coefficients:
##              Estimate Std. Error z value Pr(>|z|)
## (Intercept)  -1.72612    0.07268 -23.750  < 2e-16 ***
## residentTRUE -0.63615    0.09810  -6.484 8.91e-11 ***
## ---
## Signif. codes:  0 '***' 0.001 '**' 0.01 '*' 0.05 '.' 0.1 ' ' 1
##
## (Dispersion parameter for binomial family taken to be 1)
##
```

```
##     Null deviance: 3012.2  on 4402  degrees of freedom
## Residual deviance: 2971.0  on 4401  degrees of freedom
##   (3036 observations deleted due to missingness)
## AIC: 2975
##
## Number of Fisher Scoring iterations: 5
```

```
exp(summary(mod_oral_trainee)$coefficients[2,1] +
            qnorm(c(0.025,0.5,0.975)) * summary(mod_oral_trainee)$coefficients[2,2])
```

```
## [1] 0.4367333 0.5293257 0.6415486
```

Logistic regression adjusted for type of submission and trainee status

```
mod_oral <- glm(oral ~ sex + type_2 + resident, data = amcg,
                family = binomial)

summary(mod_oral)
```

```
##
## Call:
## glm(formula = oral ~ sex + type_2 + resident, family = binomial,
##     data = amcg)
##
## Deviance Residuals:
##     Min       1Q   Median       3Q      Max
## -1.0525  -0.0816  -0.0729  -0.0729   3.4652
##
## Coefficients:
##                            Estimate Std. Error z value Pr(>|z|)
## (Intercept)                -5.70446    0.34338 -16.613   <2e-16 ***
## sexFemale                  -0.07205    0.13902  -0.518   0.6043
## type_2Original research     5.40331    0.33925  15.927   <2e-16 ***
## residentTRUE               -0.22484    0.11850  -1.897   0.0578 .
## ---
## Signif. codes:  0 '***' 0.001 '**' 0.01 '*' 0.05 '.' 0.1 ' ' 1
##
## (Dispersion parameter for binomial family taken to be 1)
##
##     Null deviance: 3012.2  on 4402  degrees of freedom
## Residual deviance: 1714.9  on 4399  degrees of freedom
##   (3036 observations deleted due to missingness)
## AIC: 1722.9
##
## Number of Fisher Scoring iterations: 8
```

adjusted odds ratio for sex(female) and 95% CI

```
exp(summary(mod_oral)$coefficients[2,1] +
            qnorm(c(0.025,0.5,0.975)) * summary(mod_oral)$coefficients[2,2])
```

```
## [1] 0.7085642 0.9304886 1.2219203
```

adjusted odds ratio for type of submission (original research) and 95% CI

```
exp(summary(mod_oral)$coefficients[3,1] +
          qnorm(c(0.025,0.5,0.975)) * summary(mod_oral)$coefficients[3,2])
```

```
## [1] 114.2495 222.1398 431.9150
```

adjusted odds ratio for trainee status(resident = TRUE) and 95% CI

```
exp(summary(mod_oral)$coefficients[4,1] +
          qnorm(c(0.025,0.5,0.975)) * summary(mod_oral)$coefficients[4,2])
```

```
## [1] 0.6331183 0.7986404 1.0074365
```

log-binomial model for adjusted relative risks

```
lb_mod <- glm(oral ~ sex + type_2 + resident, data = amcg,
              family=binomial(link="log"))

summary(lb_mod)
```

```
##
## Call:
## glm(formula = oral ~ sex + type_2 + resident, family = binomial(link = "log"),
##     data = amcg)
##
## Deviance Residuals:
##     Min       1Q   Median       3Q      Max
## -1.0541  -0.0790  -0.0738  -0.0738   3.4519
##
## Coefficients:
##                            Estimate Std. Error z value Pr(>|z|)
## (Intercept)                -5.77048    0.33660 -17.143   <2e-16 ***
## sexFemale                  -0.04995    0.08716  -0.573    0.567
## type_2Original research     4.91781    0.33493  14.683   <2e-16 ***
## residentTRUE               -0.13733    0.07215  -1.903    0.057 .
## ---
## Signif. codes:  0 '***' 0.001 '**' 0.01 '*' 0.05 '.' 0.1 ' ' 1
##
## (Dispersion parameter for binomial family taken to be 1)
##
##     Null deviance: 3012.2  on 4402  degrees of freedom
## Residual deviance: 1714.9  on 4399  degrees of freedom
##   (3036 observations deleted due to missingness)
## AIC: 1722.9
##
## Number of Fisher Scoring iterations: 8
```

```
exp(coef(lb_mod))
```

```
##             (Intercept)                sexFemale type_2Original research
##            3.118259e-03             9.512778e-01            1.367032e+02
##            residentTRUE
##            8.716846e-01
```

adjusted relative risk for sex(female) and 95% CI

```r
exp(summary(lb_mod)$coefficients[2,1] +
            qnorm(c(0.025,0.5,0.975)) * summary(lb_mod)$coefficients[2,2])
```

```
## [1] 0.8018946 0.9512778 1.1284893
```

adjusted relative risk for type of submission (original research) and 95% CI

```r
exp(summary(lb_mod)$coefficients[3,1] +
            qnorm(c(0.025,0.5,0.975)) * summary(lb_mod)$coefficients[3,2])
```

```
## [1]  70.90673 136.70320 263.55417
```

adjusted relative risk for trainee status (resident = TRUE) and 95% CI

```r
exp(summary(lb_mod)$coefficients[4,1] +
            qnorm(c(0.025,0.5,0.975)) * summary(lb_mod)$coefficients[4,2])
```

```
## [1] 0.7567375 0.8716846 1.0040919
```
