# Supplementary material for: Role of Female Research at the Asociacion Mexicana de Cirugia General Annual Meeting: A Retrospective Analysis From 2013 to 2019
Source: Front Surg. 2022 May 13;9:900076. doi: 10.3389/fsurg.2022.900076 (PMC9406406; doi:10.3389/fsurg.2022.900076)
Supplement: Supplementary file 2 [file Table_1_v1.docx]

Supplementary Material

# Supplementary Table

## Supplementary table 1.

| **Variable** | **Univariate analysis** |  | **Multivariate analysis** |  |
| --- | --- | --- | --- | --- |
|  | **OR (95% CI)** | ***p* value** | **aRR (95% CI)** | ***p* value** |
| *Sex* |  |  |  |  |
| Male | Reference | – | Reference | – |
| Female | 0.76 (0.63-0.91) | 0.003 | 0.95 (0.80-1.12) | 0.6 |
| *Type of research* |  |  |  |  |
| Case report | Reference | – | Reference | – |
| Original research | 368.71 (202.71-670.65) | <0.001 | 136.70 (70.90-263.55) | <0.001 |
| *Surgery trainee* |  |  |  |  |
| No | Reference | – | Reference | – |
| Yes | 0.52 (0.43-0.64) | <0.001 | 0.87 (0.75-1.00) | 0.058 |

OR: odds ratio; aRR: adjusted relative risk

**Supplementary table 1.** Univariate logistic regression and multivariate log-binomial regression model for selection as oral presentation.

## Supplementary table 2.

| **Variable** | **Univariate analysis** |  | **Multivariate analysis** |  |
| --- | --- | --- | --- | --- |
|  | **OR (95% CI)** | ***p* value** | **aRR (95% CI)** | ***p* value** |
| *Sex* |  |  |  |  |
| Male | Reference | – | Reference | – |
| Female | 0.75 (0.62-0.89) | 0.002 | 0.90 (0.68-1.20) | 0.49 |
| *Type of research* |  |  |  |  |
| Case report | Reference | – | Reference | – |
| Original research | 381.01 (209.20-693.92) | <0.001 | 235.50 (120.97-458.46) | <0.001 |
| *Surgery trainee* |  |  |  |  |
| No | Reference | – | Reference | – |
| Yes | 0.50 (0.41-0.61) | <0.001 | 0.71 (0.55-0.91) | 0.006 |

OR: odds ratio; aRR: adjusted relative risk

**Supplementary table 2.** Univariate logistic regression and multivariate log-binomial regression model for selection as oral presentation excluding rejected abstracts.

# Supplementary Figures

## Supplementary Figure 1

**Supplementary Figure 1.** Medical school graduates achieving a passing score in the National Examination for Medical Residency Candidates (ENARM) applying to Mexican surgery programs from 2013 to 2019.
